# Trait‐based approaches to analyze links between the drivers of change and ecosystem services: Synthesizing existing evidence and future challenges

**DOI:** 10.1002/ece3.2692

**Published:** 2017-01-04

**Authors:** Violeta Hevia, Berta Martín‐López, Sara Palomo, Marina García‐Llorente, Francesco de Bello, José A. González

**Affiliations:** ^1^ Social‐ecological Systems LaboratoryDepartment of EcologyUniversidad Autónoma de MadridMadridSpain; ^2^ Faculty of SustainabilityInstitute of Ethics and Transdisciplinary Sustainability ResearchLeuphana University of LüneburgLüneburgGermany; ^3^ Applied Research and Agricultural Extension DepartmentMadrid Institute for Rural, Agricultural and Food Research and Development (IMIDRA)Alcalá De HenaresMadridSpain; ^4^ Institute of BotanyAcademy of Sciences of the Czech RepublicTrebonCzech Republic

**Keywords:** biodiversity, ecosystem function, effect traits, global environmental change, response traits, systematic review

## Abstract

Understanding the responses of biodiversity to drivers of change and the effects of biodiversity on ecosystem properties and ecosystem services is a key challenge in the context of global environmental change. We performed a systematic review and meta‐analysis of the scientific literature linking direct drivers of change and ecosystem services via functional traits of three taxonomic groups (vegetation, invertebrates, and vertebrates) to: (1) uncover trends and research biases in this field; and (2) synthesize existing empirical evidence. Our results show the existence of important biases in published studies related to ecosystem types, taxonomic groups, direct drivers of change, ecosystem services, geographical range, and the spatial scale of analysis. We found multiple evidence of links between drivers and services mediated by functional traits, particularly between land‐use changes and regulating services in vegetation and invertebrates. Seventy‐five functional traits were recorded in our sample. However, few of these functional traits were repeatedly found to be associated with both the species responses to direct drivers of change (response traits) and the species effects on the provision of ecosystem services (effect traits). Our results highlight the existence of potential “key functional traits,” understood as those that have the capacity to influence the provision of multiple ecosystem services, while responding to specific drivers of change, across a variety of systems and organisms. Identifying “key functional traits” would help to develop robust indicator systems to monitor changes in biodiversity and their effects on ecosystem functioning and ecosystem services supply.

## 1 Introduction

Global biodiversity is being severely affected by drivers of change that are directly or indirectly induced by human activities. Direct drivers of change include land‐use change, climate change, invasive alien species, overexploitation, and pollution (Pereira, Navarro, & Martins, 2012; Vitousek, Mooney, Lubchenco, & Melillo, 1997). The loss of biodiversity may alter ecosystem functioning and the delivery of ecosystem services, with major repercussions on human well‐being (Balvanera et al., 2006; Dirzo et al., 2014; Hanski et al., 2012; Mace, Norris, & Fitter, 2012). Although biodiversity is assumed to be critical for providing ecosystem services (Cardinale et al., 2012; De Bello et al., 2010; Harrison et al., 2014), our understanding about the links between biodiversity and individual ecosystem services remains incomplete (Balvanera et al., 2014; Bennett et al., 2015; Isbell et al., 2011; Suding et al., 2008). Lavorel et al. (2007) suggested that understanding the responses of biodiversity to drivers and the effects of biodiversity on ecosystem services is critical for developing future scenarios about the effects of global environmental change. Yet, our knowledge about the linkages between specific drivers of change and ecosystem properties modulated by biodiversity remains limited.

It has become increasingly clear that both the responses of biodiversity to drivers of change and the effects of biodiversity on ecosystem services may be explained by functional traits (Díaz et al., 2007). Functional traits determine the organism's response to pressures and drivers of change (response traits) and its effects on ecosystem properties and the provision of ecosystem services (effect traits; Cadotte, Carscadden, & Mirotchnick, 2011; De Bello et al., 2010; Hooper et al., 2005; Valiente‐Banuet et al., 2015). Recent trait‐based approaches have assessed how ecosystem services might be affected by drivers of change (Quétier, Lavorel, Thuiller, & Davies, 2007) through the analysis of effect and response traits (Díaz et al., 2007, 2013; Lavorel, 2013; Lavorel & Garnier, 2002; Lavorel et al., 2011; Suding et al., 2008). These trait‐based approaches might prove effective for improving ecosystem management and decision‐making within the context of environmental change (Lavorel, 2013; Nagendra, Reyers, & Lavorel, 2013).

Here, we performed a systematic literature review and meta‐analysis to synthesize existing empirical evidence about the interlinkages among direct drivers of change and ecosystem services, mediated by functional traits of three taxonomic groups (vegetation, invertebrates, and vertebrates). There have been several scientific literature reviews on how the direct drivers of change are linked with functional traits (e.g., Verheyen, Honnay, Motzkin, Hermy, & Foster, 2003) or how functional traits are linked with ecosystem services (e.g., De Bello et al., 2010; Harrison et al., 2014; Ricketts et al., 2016). However, to the best of our knowledge, this work presents the first systematic review on the entire pathway, from drivers to ecosystem services via traits, across different taxonomic groups.

First, we reviewed the status and general trends in the scientific literature to characterize the “research landscape” in this field until 2014. Second, we compiled and synthesized existing evidence of relationships among drivers of change, functional traits, and ecosystem services. Then, we explored the existence of “bundles of traits” associated with particular direct drivers of change and ecosystem services. Finally, we identified existing knowledge gaps and suggested future challenges in the application of trait‐based approaches for biodiversity monitoring.

## 2 Materials and Methods

### 2.1 Literature search

We conducted a Web of Science survey up to 2014, using search terms related to functional traits (*N* = 29 terms), combined with direct drivers of change (*N* = 33 terms) and ecosystem services and all potential synonyms (*N* = 72 terms; see Appendix S1 for the complete list of the keywords used in the systematic review). We acknowledge that our search terms might include some publications that focus on ecosystem functions, ecological processes, or benefits, which, under certain definitions, would not properly qualify as “ecosystem services.” Basically, the ecosystem services concept is complex and subjected to multiple interpretations (Abson et al., 2014; Nahlik, Kentula, Fennessy, & Landers, 2012). Given that there is not yet a single, unifying definition of ecosystem services (Nahlik et al., 2012), here, we embraced the proposal of Mace et al. (2012): “an activity or function of an ecosystem that provides benefit to humans.” This definition encompasses the entire pathway from ecological processes to final ecosystem services, being the one that best fits with the approach of our review. Thus, we selected sufficiently broad enough search terms to include all ecosystem functions/services identified in the Millennium Ecosystem Assessment (MA) and the Common International Classification of Ecosystem Services (CICES; http://cices.eu/).

The literature search resulted in a sample of 302 papers, of which 125 fit the criteria for inclusion, that is, papers that have empirically used trait‐based approaches to analyze links between the drivers of change and ecosystem services. Appendix S2 shows the diagram flow of the methodological process.

### 2.2 Data collection

Following the content analysis of these selected papers, two databases were created. The first database (*N* = 125 papers; see Appendix S3, for the complete list of publications) was used to characterize the current state and trends of trait‐based ecosystem services research, including information on: (1) publication characteristics (i.e., year of publication, type of research); (2) study area; (3) methodological approach used (e.g., data source, theoretical or analytical approach); (4) taxonomic group studied; (5) ecosystem type; (6) direct drivers of change analyzed; (7) functional traits used; (8) category of ecosystem services (i.e., provisioning, regulating, or cultural); and (9) specific ecosystem services investigated. Appendix S4 summarizes the list of attributes used to characterize publications.

The second database was traits‐oriented and only considered those statistically significant relationships among drivers of change, functional traits, and ecosystem services found in the existing literature (*N* = 83 observations, from 71 papers). In this database, we codified (as dummy variables) those relationships between drivers and response traits, and/or between effect traits and ecosystem services, for those studies that reported significant evidence. As we could not incorporate any weighting of the magnitude of the responses and/or effects, we acknowledge that this might result in an overrepresentation of those functional traits that have been most frequently investigated.

### 2.3 Data analysis

To address the current status and trends of research in this field, we performed frequency analyses on ecosystem types, taxonomic groups, functional traits, direct drivers of change, and ecosystem services (using the first database). After analyzing research trends, we focused on synthesizing the existing evidence of links between drivers and ecosystem services mediated by functional traits (using the second database). In doing so, we first analyzed emerging patterns, focusing particularly on how land‐use change affects regulating services, which is the relationship that has been most extensively tested using functional traits.

To draw general conclusions from existing evidence of interlinkages between drivers of change and functional traits, as well as between functional traits and ecosystem services, we conducted six different redundancy analyses (RDAs). Three RDAs were performed to synthesize the evidence of interlinkages between direct drivers of change (used as explanatory variables) and response traits (as dependent variables) for each of the three taxonomic groups. Then, three other RDAs were performed to synthesize the existing evidence linking effect traits (used as explanatory variables) and ecosystem services (as dependent variables). In all analyses, the dependent and explanatory variables were dichotomous according to the existence of evidence about relationships between drivers of change and response traits and between effect traits and ecosystem services. A Monte Carlo permutation test (500 permutations) was performed to determine the significance of explanatory variables. RDAs were performed using XLSTAT 2012 (Addinsoft) software.

## 3 Results

### 3.1 Status and trends in trait‐based ecosystem services research

**Figure 1:**
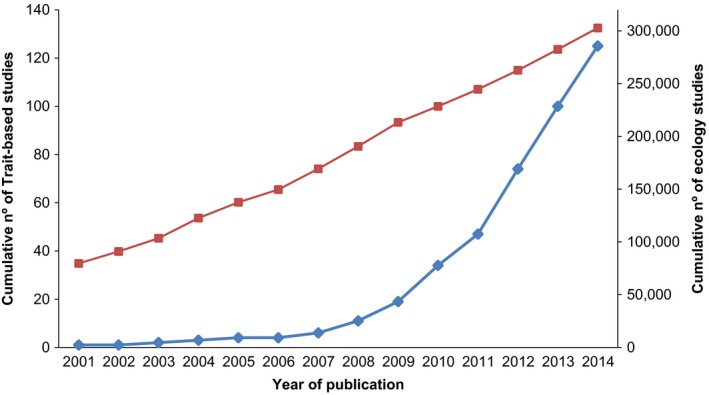
Trends in the scientific literature exploring the links among drivers of change, functional traits and ecosystem services, compared with general trends in ecology scientific literature. Blue line indicates the cumulative number of studies considered in this systematic review along our study period (*Y* axis on the left side). Red line indicates the cumulative number of ecology studies along our study period (*Y* axis on the right side). The general trend of ecology research was obtained by a survey up to 2014 in the Web of Science, using “ecology” or “ecolog*” as search terms

Temporal trends in our sample show that this topic is an emerging research field, with an exponential increase in the number of trait‐based papers that contrast with the arithmetic increase in ecology research (Figure 1). Although the first empirical trait‐based study was published in 2001 (i.e., Dukes, 2001), the number of papers grew exponentially between 2008 (*N* = 5) and 2012 (*N* = 27), but plateaued during 2013 and 2014.

**Figure 2:**
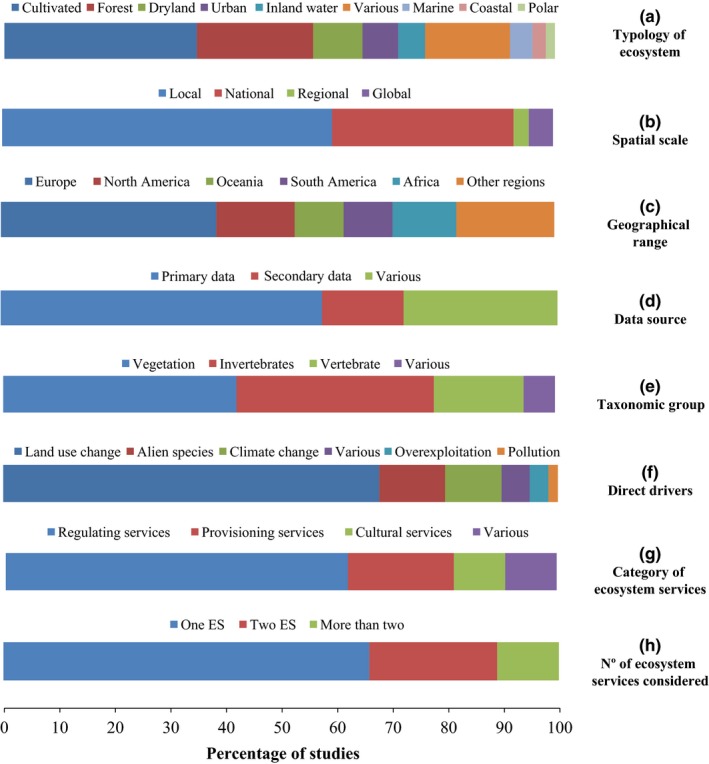
Characterization of the peer‐reviewed literature sample (*N* = 125) according to the percentage of studies: (a) conducted on each type of ecosystem; (b) conducted at different spatial scales; (c) conducted at different geographical regions; (d) using different data sources; (e) focusing on each taxonomic group; (f) analyzing each direct driver of change; (g) analyzing each category of ecosystem services; and (h) according to the number of ecosystem services considered

Most publications corresponded to cultivated agroecosystems (35.9%), forests (21.1%), and dryland ecosystems (11.0%; Figure 2A). Most studies were conducted at a local (60.3%) or national (34.0%) scale, with very few being conducted at regional or global scales (Figure 2b). Most of the research was conducted in Europe (38.9%), followed by North America and Oceania (14.1% and 8.8%, respectively; Figure 2c). Most studies in our sample (57.7%) were based on primary data, while the remainder used secondary sources (14.6%) or a mix of both data types (27.6%; Figure 2d). Vegetation and invertebrates (i.e., insects) were the most studied taxonomic groups (40.4% and 37.4% of the sampled papers, respectively), with research on vertebrates being scarcer (16.6%; Figure 2e).

Land‐use change was the most frequently studied driver of change in our sample, with 67.8% of the studies only focusing on analyzing this specific driver and its effects. Studies on invasive alien species and climate change were also relevant in our sample (11.8% and 10.1%, respectively). In contrast, the interlinkages between other drivers, such as pollution or overexploitation, and ecosystem services via functional traits have been rarely examined. Only five studies were recorded that simultaneously analyzed the effect of various drivers of change (Figure 2f).

Most studies focused on exploring regulating services (62.1%), followed by provisioning services (19.2%), whereas studies on cultural services were scarce (9.3%). Again, few studies simultaneously assessed more than one category of ecosystem services (Figure 2g). Finally, most papers investigated only one (65.8%) or two ecosystem services (23.0%), with just 11.1% of studies assessing more than two ecosystem services (Figure 2h).

**Figure 3:**
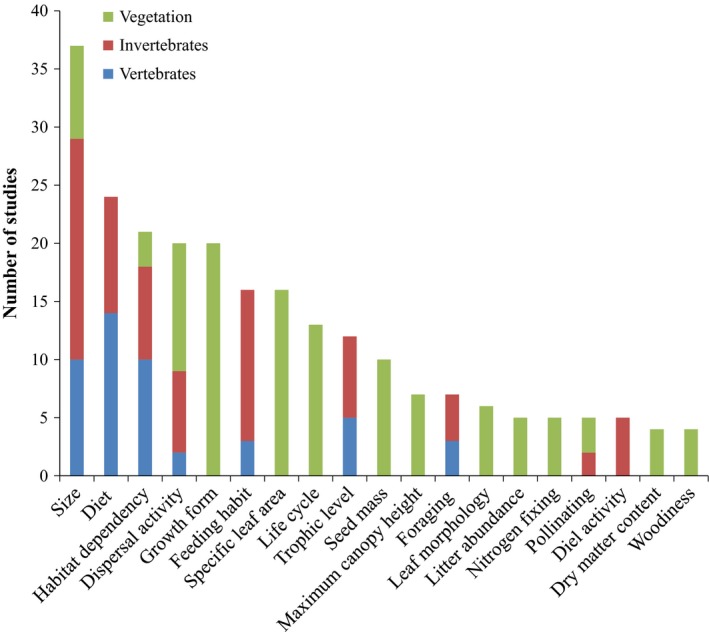
Number of studies using each of the most frequently analyzed functional traits (only those traits used in more than three papers are represented) in the scientific literature for the three taxonomic groups

A total of 75 functional traits were recorded in our dataset: 41 for vegetation, 25 for invertebrates, and 20 for vertebrates (Appendix S5). The most frequently investigated trait was size, which was used for all three analyzed taxonomic groups. The next most frequently investigated trait was diet for vertebrates and invertebrates, followed by habitat dependency (mostly for vertebrates and invertebrates), dispersal activity (for all three groups), and growth form (for vegetation; Figure 3).

### 3.2 Research linking direct drivers of change, functional traits, and ecosystem services

**Figure 4:**
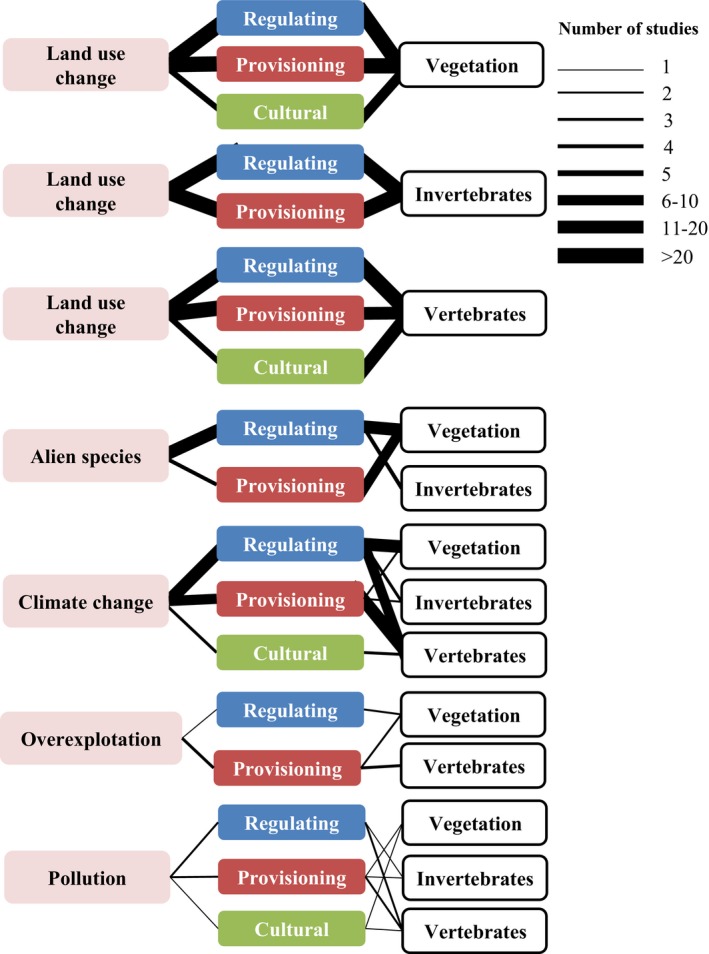
Number of studies in the sample that empirically explored the impacts of the drivers of change on ecosystem services mediated by the functional traits of each of the three taxonomic groups. In the case of land‐use change, the links are presented separately for each taxonomic group, to facilitate figure readability

The relationships between land‐use change and regulating services were clearly most frequently addressed using a trait‐based approach (73.6% of the papers; Figure 4), particularly for links mediated by vegetation and invertebrate traits. Among regulating services potentially affected by land‐use change via functional traits, habitat provision, pest control, and nutrient cycling were the most analyzed. Relationships of land‐use change with provisioning services have also been largely explored in the published literature (28.0% of the papers), particularly with respect to food provision via vegetation and invertebrate traits.

After land‐use change, climate change and invasive alien species were the drivers that received the most attention in the scientific literature. Studies on the links between alien species and regulating and provisioning services mainly focused on invasion resistance mediated by vegetation traits. The scientific literature mostly explored the impacts of climate change on provisioning and regulating services, particularly those mediated by vertebrate traits (Figure 4).

Our results show that few studies have focused on how overexploitation affects provisioning services mediated by vertebrate traits (particularly of fish) or regulating services, such as invasion resistance, mediated by plant traits. Studies exploring the relationship between pollution and ecosystem services are also limited and mostly focused on the effects of water pollution on food production mediated by vertebrate traits (Figure 4).

### 3.3 Synthesizing evidence of links among drivers of change, functional traits, and ecosystem services

**Figure 5:**
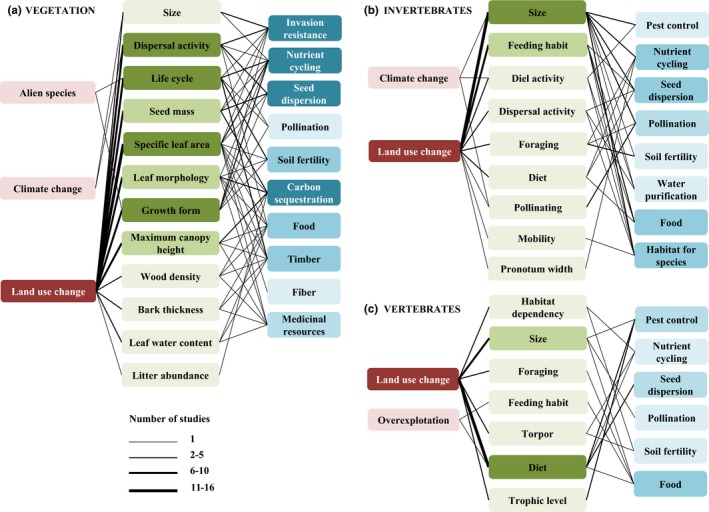
Functional traits for which empirical evidence has been found of links with drivers of change (acting as response traits) and with ecosystem services (acting as effect traits) for all three taxonomic groups. Line width indicates the number of studies reporting significant results for that relationship. Red boxes refer to the drivers of change, green boxes to the functional traits, and blue boxes to the ecosystem services. Box color intensity increases according to the number of studies reporting significant links with that variable.

Twelve vegetation traits were found to respond to land‐use change and influence six regulating services and four provisioning services. Two vegetation traits were also found to respond to climate change, while another two vegetation traits responded to alien species (Figure 5a). For invertebrates, nine traits were found to respond to land‐use change, while three traits responded to climate change. These traits were found to affect seven regulating services and one provisioning service (Figure 5b). For vertebrates, six traits were found to respond to land‐use change, while two traits responded to overexploitation. These traits affected five regulating services and one provisioning service (Figure 5c).

Overall, 84.2% of the traits analyzed acted both as response and effect traits: specifically, 90.4% for vegetation, 75.0% for invertebrates, and 87.5% for vertebrates (Figure 5). The most frequent vegetation traits that showed significant links with land‐use change and ecosystem services were size, dispersal activity, specific leaf area, life cycle, seed mass, nitrogen fixing, leaf morphology, growth form, maximum canopy height, and woodiness. All of these traits acted as both response traits to land‐use change and effect traits on certain regulating services, such as nutrient cycling and soil fertility (Table 1). In the case of invertebrates, size and feeding habit were the most common traits showing significant relationships with land‐use change. These traits also influenced several regulating services (Table 1), such as water purification and seed dispersion, acting as both response and effect traits. For vertebrates, not enough studies were available to derive any clear conclusion, although size, diet, foraging, and habitat dependency appeared to be affected by land‐use change. These traits influenced certain regulating services, such as pest control and pollination (in the case of size) and seed dispersion (in the case of diet).

**Table 1:** Number of studies that found a relationship between land‐use change and ecosystem services via functional traits (specifying, for each trait, the number of cases (*N*) where it acts as response or effect trait). Only those traits with two or more cases have been presented. For the complete list of traits and the number of studies, see Appendix S5 (SLA: specific leaf area)

Taxa	Traits	Response trait (*N*)	Effect traits (*N*)	Ecosystem service	Study type
Vegetation	Size	2	1	Nutrient cycling	Obs
			1	Invasion resistance	Obs
	Dispersal activity	6	1	Invasion resistance	Obs
			1	Seed dispersion	Obs
			1	Nutrient cycling	Obs
			1	Pollination	Obs
	SLA	9	3	Nutrient cycling	Obs
			1	Soil fertility	Pred
			1	Seed dispersion	Obs
			3	Raw materials	Obs
			1	Carbon cycling	Obs
			1	Medicinal resources	Obs
	Life cycle	7	3	Nutrient cycling	Obs
			2	Soil fertility	Pred
			1	Pollination	Obs
	Seed mass	6	1	Invasion resistance	Obs
			2	Nutrient cycling	Obs
			2	Seed dispersion	Obs
			1	Carbon cycling	Obs
	Nitrogen fixing	2	1	Nutrient cycling	Obs
			1	Raw materials	Obs
	Leaf morphology	3	1	Carbon cycling	Obs
			2	Raw materials	Obs
			1	Nutrient cycling	Obs
			1	Soil fertility	Obs
			1	Medicinal resources	Obs
	Growth form	2	2	Nutrient cycling	Obs
			1	Soil fertility	Obs
	Maximum canopy height	3	2	Carbon cycling	Obs
			2	Raw material	Obs
			1	Medicinal resources	Obs
	Woodiness	3	1	Carbon cycling	Obs
			2	Raw materials	Obs
			1	Medicinal resources	Obs
Invertebrates	Size	9	2	Soil fertility	Obs
			2	Seed dispersion	Obs
			3	Pest control	Obs
			2	Nutrient cycling	Obs
			1	Water purification	Obs
			2	Pollination	Obs
			1	Waste treatment	Obs
	Feeding habit	4	1	Water purification	Obs
			1	Seed dispersion	Obs
			1	Food	Obs
			1	Habitat for species	Obs
	Diet	2	2	Nutrient cycling	Obs
			2	Seed dispersion	Obs
	Foraging	3	1	Nutrient cycling	Obs
			1	Soil fertility	Obs
			1	Pollination	Obs
	Dispersal activity	3	1	Habitat for species	Obs
			1	Water purification	Obs
			1	Seed dispersion	Obs
Vertebrates	Size	2	1	Pest control	Obs
			1	Nutrient cycling	Obs
			1	Soil fertility	Obs
			1	Pollination	Obs
			1	Cultural services^a^	Obs
	Diet	5	3	Seed dispersion	Obs
			1	Pest control	Obs
			1	Nutrient cycling	Obs
			1	Soil fertility	Obs
	Foraging	2	1	Nutrient cycling	Obs
			1	Soil fertility	Obs
	Habitat dependency	3	1	Seed dispersion	Obs
			1	Nutrient cycling	Obs
			1	Soil fertility	Obs

a Cultural services are not specified due to few studies that analyze these ecosystem services in our review, so its interpretation would be very complex.

### 3.4 Uncovering bundles of traits associated with particular direct drivers of change and ecosystem services

**Figure 6:**
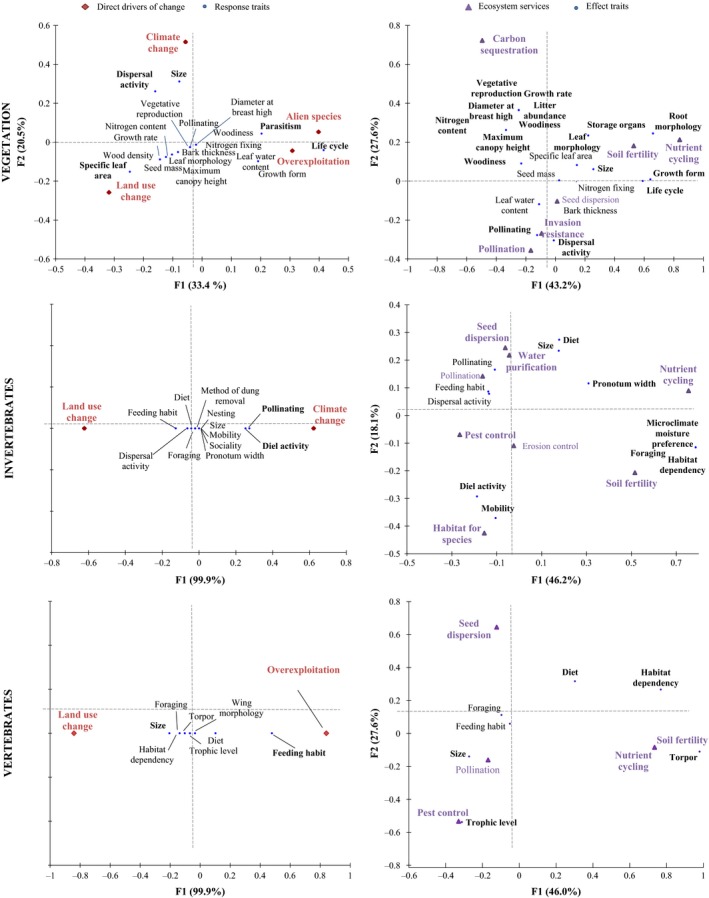
Biplots resulting from the RDAs performed for each taxonomic group to uncover the relationships between the direct drivers of change and response traits and between the effect traits and regulating services. Provisioning and cultural services are not used in this analysis as they were scarcely represented in our sample. Bold red text represents the direct drivers of change with higher standardized canonical coefficients, and bold violet text represents the ecosystem services with higher squared cosines for axes 1 and 2. Bold black font represents the response traits with higher squared cosines, while for the effect traits, bold black font represents the traits with higher standardized canonical coefficients

RDAs of the relationship between direct drivers of change and response traits revealed different bundles for each taxonomic group (Figure 6; Appendix S6). For vegetation, land‐use change was related to specific leaf area in the negative F1 scores, while alien species and overexploitation were related to life cycle and parasitism in the positive scores. In F2, climate change was related to size and dispersal activity in the positive scores (Figure 6).

For invertebrates, climate change appeared to be strongly related to diel activity and pollinating in the positive F1 scores (Figure 6). For vertebrates, land‐use change was related to size (negative F1 scores), while overexploitation was related to feeding habit (positive F1 scores; Figure 6).

RDAs of the relationship between effect traits and ecosystem services also showed different bundles for each taxonomic group (Figure 6; Appendix S7). For vegetation, positive F1 scores showed relationships between size, leaf morphology, life cycle, storage organs, root morphology, and growth form with soil fertility and nutrient cycling (Figure 6). Many vegetation effect traits (litter abundance, maximum canopy height, woodiness, vegetative reproduction, growth rate, nitrogen content, and diameter at breast height) had negative F1 scores and positive F2 scores related to carbon cycling. Negative F2 scores for pollinating were related to pollination service and invasion resistance (Figure 6).

For invertebrates, positive F1 scores showed a bundle of different effect traits (pronotum width, diet, size, habitat dependency, foraging, and microclimate moisture preference) with nutrient cycling and soil fertility. Negative F2 scores were obtained for diel activity and mobility linked with habitat for species, whereas positive F2 scores were obtained for size and diet related to seed dispersion and water purification (Figure 6).

The specific RDA for vertebrates revealed a relationship between diet, habitat dependency, and torpor with nutrient cycling and soil fertility in the positive F1 scores. In the negative F1 scores, size and trophic level relate to pest control and seed dispersion. Diet was related to seed dispersion in the positive F2 scores (Figure 6).

## 4 Discussion

Our literature review documents existing evidence of links between the direct drivers of change and the supply of ecosystem services, mediated by the functional traits that modulate how species respond to drivers and how they affect ecosystem properties. We acknowledge that our results mostly reflect what has been studied to date, rather than the intensity and degree of those significant relationships. However, the evidence synthesized here may help improve our understanding about the linkages between the response of biodiversity to environmental change and biodiversity effects on ecosystem services, which is the missing link of the so‐called holy grail in functional ecology (Lavorel & Garnier, 2002; Lavorel et al., 2007).

### 4.1 Gaps and biases in trait‐based approaches to analyze links between drivers and ecosystem services

Our results on the historical trends in functional traits‐ecosystem services investigation are consistent with previous studies that analyzed the temporal evolution of general ecosystem services research in different ecoregions and at different geographical scales (Nieto‐Romero, Oteros‐Rozas, González, & Martín‐López, 2014; Vihervaara, Rönkä, & Walls, 2010). However, in contrast to previous studies (Vihervaara et al., 2010), we found that trait‐based research is clearly biased toward agroecosystems (mostly cultivated areas) and forest ecosystems, whereas studies on inland aquatic, coastal, and marine systems remain limited. Our review also shows some biases in the geographical coverage of studies, with important gaps existing in the tropical regions of South America, Africa, and Southeast Asia, which are essential for global biodiversity conservation (Myers, Mittermeier, Mittermeier, da Fonseca, & Kent, 2000). Most of the studies were conducted in Europe, which is coherent with the extended application of the ecosystem services approach in this region (Seppelt, Dormann, Eppink, Lautenbach, & Schmidt, 2011). This geographical bias is particularly relevant given that the influence of traits in ecosystem functioning and the provision of ecosystem services are highly context dependent (Abelleira‐Martínez et al., 2016; Hooper et al., 2005; Srivastava & Vellend, 2005). Consequently, this bias largely hinders the global application of trait‐based approaches at present.

Furthermore, this review showed a clear bias toward research conducted at local scales. The spatial scale of the analysis has a strong influence on the form of the relationship among land‐use change, functional traits, and ecosystem services (Gross, Willig, Gough, Inouye, & Cox, 2000; Hevia et al., 2016). Consequently, it is important to address how the scale of land‐use activities affects functional traits and how this might affect the provision of ecosystem services at multiple scales (De Lima, Dallimer, Atkinson, & Barlow, 2013; Gilroy, Medina‐Uribe, Haugaasen, & Edwards, 2015; Nagendra et al., 2013; Newbold et al., 2014).

Interestingly, few papers studied various groups of organisms simultaneously in the trait‐based literature. To overcome this important gap, there have been recent calls for cross‐taxon studies (Moretti et al., 2013) and for the use of functional metrics across trophic levels to develop more comprehensive biodiversity monitoring (Hevia et al., 2016; Lavorel et al., 2013; Vandewalle et al., 2010).

Most trait‐based studies have focused on the effects of land use (Figure 4), which is coherent because land‐use change is the most important direct driver of biodiversity erosion at a global scale (Pereira et al., 2012). Thereby, it has received more scientific attention than any other driver of change in biodiversity conservation literature (Fazey, Fischer, & Lindenmayer, 2005; Velasco et al., 2015). In particular, recent studies have demonstrated how land‐use intensification is related to the loss of functional traits and erosion of multiple ecosystem services (Brown et al., 2013; García‐Llorente et al., 2015; Laliberté et al., 2010).

Similar to what has been found for drivers of change, few studies have assessed more than one category of ecosystem services simultaneously. These findings are consistent with previous reviews showing that regulating services are the category receiving the greatest focus in ecological research (Harrison et al., 2014). This result may be explained by the evident direct link between regulating services and ecosystem functions, which is less distinct for other service categories (i.e., provisioning and cultural services) that are more dependent on social constructs (Daniel et al., 2012). Recent studies have also highlighted that functional traits more closely related to cultural ecosystem services are those that receive less attention (e.g., organism color, birdsong, and olfactory traits; Goodness, Andersson, Anderson, & Elmqvist, 2016). Therefore, additional studies are required to assess the potential effects of drivers of change on cultural or provisioning services, via less‐conventional functional traits.

Furthermore, most studies in this review only investigated one ecosystem service, which is consistent with previous reviews of ecosystem services research (Mitchell et al., 2013; Nieto‐Romero et al., 2014; Seppelt et al., 2011). The fact that the functional trait literature has not addressed multiple ecosystem services largely hinders its potential application in landscape management, as this application necessarily requires uncovering ecosystem services trade‐offs and synergies (i.e., negative and positive associations between ecosystem services, respectively; Mouchet et al., 2014).

### 4.2 Searching for key functional traits linking drivers and ecosystem services

We found that some single functional traits (e.g., size or diet) may contribute to the provision of several ecosystem services, while responding to specific drivers of change (e.g., land‐use change and climate change; see Figure 4). This indicates their potential role as “key functional traits,” involved in the regulation of the system. “Keystone species” refer to specific system elements able to guarantee ecosystem functioning and the provision of multiple ecosystem services (Biggs et al., 2012). Thus, here we propose that specific functional traits that influence the provision of diverse ecosystem services and respond to drivers of change across a variety of systems and organisms might be considered as “key functional traits.” In fact, these are traits that, if affected by a given driver of change, will have major consequences on ecosystem functioning. Therefore, it could be effective to focus environmental monitoring efforts on these traits, because of their potential effects on multiple ecosystem properties and services. Further, as some of these key functional traits (e.g., size) are relevant for different taxonomic groups, they might also be useful for incorporating cross‐taxon and multitrophic perspectives to this research topic (Lavorel, 2013).

Establishing relationships among direct drivers of change, key functional traits and ecosystem services could lead to a major advance in ecological research (Lavorel & Garnier, 2002). Our review suggests that an improved understanding about the key functional traits, associated with both the capacity to respond to environmental changes and the capacity to contribute to ecosystem properties, could help develop robust indicator systems to monitor changes in biodiversity and their effect on ecosystem functioning and the delivery of ecosystem services. Some of the identified key functional traits are relatively easy to measure (e.g., size, leaf morphology), making them particularly useful for monitoring the effects of environmental change on ecosystem properties and the potential supply of ecosystem services. In this sense, the identification of the key functional traits can contribute to the further development of the essential biodiversity variables (EBVs; Pereira et al., 2013) within the EBV class of species traits. Further, such knowledge might be also relevant for the global and regional biodiversity and ecosystem services assessments that have been recently launched by the Intergovernmental Platform of Biodiversity and Ecosystem Services (IPBES), because the trait‐based approach shows the importance of particular traits for mediating between direct drivers of change and the supply of “*nature's benefits to people”* (Díaz et al., 2015). Thus, the present study could contribute to both initiatives, EBVs and IPBES, by providing a synthesis of evidence that has already been published.

To date, few studies have tested the overlap between response and effect traits that actually underlie the relationships between drivers and ecosystem services (but see Díaz et al., 2013; Suding et al., 2008). While more studies are certainly needed in this direction, our results provide indirect but novel evidence of this type of overlap. Our analyses suggest that most response traits that are strongly associated with specific direct drivers of change also act as effect traits. Although this is just a preliminary indication of the strength of the overlap between response and effect traits, our results suggest that the same traits studied in response to environmental change across a variety of systems and organisms may be involved in the control of ecosystem function and the supply of particular ecosystem services. This finding might have important implications for the resilience of ecosystems in the face of environmental change (Nimmo, Mac Nally, Cunningham, Haslem, & Bennett, 2015; Seidl et al. 2015); Suding et al., 2008 and, thereby, for the resilience of associated ecosystem services (Biggs, Schlüter, & Schoon, 2015; Biggs et al., 2012; Díaz et al., 2013). The overlap between effect and response traits may lead to different resilience pathways in the community (Oliver et al., 2015). If there is a positive correlation between effect and response traits, a decline in the populations of species with those traits after a particular environmental perturbation may lead to a decline in the ecological properties fostered by particular effect traits that appear in such populations. For example, the trait of body size in female bees acts as a response trait under agricultural intensification, but also acts as an effect trait that contributes to pollination efficiency. This correlation between effect and response traits may lead to a decline in the ecosystem service of pollination following agricultural intensification processes (Larsen, Williams, & Kremen, 2005).

In contrast, completely uncorrelated response and effect traits may guarantee the maintenance of ecological properties when the responses of species to environmental perturbations are decoupled from their effects on ecological processes (Díaz et al., 2013; Oliver et al., 2015). For example, Radchuk, Laender, Brink, and Grimm (2015) found that insecticides in freshwater systems affect particular feeding guilds (response trait) of zooplankton (i.e., herbivores, carnivores, and detritivores), but this does not destabilize the ecological processes of gross primary production and respiration. The main reason is that effect traits that seem to foster both ecological processes are different traits, such as body size and the feeding guild of omnivores. This example also pinpoints that the provision of ecosystem services often depends on the interactions between multiple traits across multiple trophic levels (Lavorel et al., 2013; Thompson, Davies, & Gonzalez, 2015).

Finally, an overlap between effect and response traits shows that species that have similar contributions to a particular ecological process may differ in their responses to disturbances and, thereby, might enhance the resilience of the system by increasing response diversity (Mori, Furukawa, & Sasaki, 2013; Suding et al., 2008). For instance, seed dispersion in Uganda forests is performed by mammals with a diverse range of sizes, from mice to chimpanzees. Under localized disturbances, such as land‐use change, small mammals with low mobility are negatively affected, whereas more mobile and larger species maintain the seed dispersal function (Peterson, Allen, & Holling, 1998). However, it is important to note that the overlap between effect and response traits is only one of the mechanisms that enhance the resilience of ecosystem services. Many other mechanisms have been identified in the literature, such as genetic variability, species diversity, species populations, landscape heterogeneity, and landscape functional connectivity (Biggs et al., 2015; Nimmo et al., 2015; Oliver et al., 2015).

### 4.3 Future challenges in trait‐based ecosystem services research

Despite trait‐based ecosystem services research having developed considerably over the last decade, our scientific understanding about the interlinkages among direct drivers of change and ecosystem services mediated by functional traits remains limited. Based on the biases found in our review, we propose here three major challenges for future research: (1) expanding spatial scales and geographical coverage; (2) addressing complex relationships through cross‐taxon, multitrophic approaches; and (3) addressing associations and interactions among functional traits.

First, despite recent advances, additional research is needed to fill current knowledge gaps, particularly with respect to several types of ecosystems, geographical coverage and the scale of analysis. For example, more research is needed to identify particular characteristics in the relationships among drivers, traits, and ecosystem services in currently less‐studied ecosystems (e.g., inland aquatic, coastal, and marine systems) and geographical regions (e.g., tropical areas). Moreover, although the trait‐based approach has been validated at local scales (Lavorel et al., 2013), certain drivers of change (such as climate change) operate at much broader scales. Thus, the trait‐based approach should also be applied beyond the local scale (Wood et al., 2015).

Second, although research within the last few years has begun to use a multitrophic approach, by considering the interaction between vegetation traits and other organisms' traits (Grigulis et al., 2013; Lavorel et al., 2013; Moretti et al., 2013; Storkey et al., 2013), it is important to further characterize traits across taxonomic groups and trophic levels, as well as their interrelationships (Lavorel, 2013; Violle, Reich, Pacala, Enquist, & Kattge, 2014; Wood et al., 2015). To develop these cross‐taxon and multitrophic trait‐based approaches, it might be crucial to be able to use a shared code of traits. Furthermore, such cross‐taxon comparison would require improving collaborative data sharing. This could be facilitated by the development of trait databases, such as TRY (http://www.try-db.org/, Kattge et al., 2011) and TraitNet (http://raitnet.ecoinformatics.org/) that have been developed for plants at a global scale. Trait databases also exist for animals at a regional scale, including vertebrates (i.e., fish; Frimpong & Angermeier, 2009) and invertebrates, such as ground beetles (Homburg, Homburg, Schäfer, Schuldt, & Assmann, 2014), cavity‐nesting wasps and bees (Scales project; http://www.scales-project.net/), hoverflies (Speight, Castella, & Sarthou, 2013), and aquatic macroinvertebrates (Statzner, Bonada, & Dolédec, 2008; Vieira et al., 2006). However, for most taxonomic groups of invertebrates and vertebrates, available trait databases are still missing (Gossner et al., 2015).

Finally, we found that most functional traits that are responsible for the response of species to various direct drivers of change (response traits) are also traits that affect ecosystem services supply (effect traits). The multivariate analyses allowed us to identify some key functional traits, which were delineated as those that have the potential capacity to provide multiple ecosystem services while responding to specific drivers of change. Future research to consolidate a list of traits (and bundles of traits) that are able to respond to drivers of change, while maintaining the provision of ecosystem services, would be highly relevant to design and apply robust environmental policies that ensure the conservation of these “key functional traits” and, thereby, preserve the resilience of ecosystems.

## Conflict of Interest

None declared.
