# Supplementary material for: Trait‐based approaches to analyze links between the drivers of change and ecosystem services: Synthesizing existing evidence and future challenges
Source: Ecol Evol. 2017 Jan 4;7(3):831–44. doi: 10.1002/ece3.2692 (PMC5288245; doi:10.1002/ece3.2692)
Supplement: Supplementary file 1 [file ECE3-7-831-s001.doc]

**Appendix S1. List of all of the keywords used in the systematic review:**

**Direct Drivers of Change**

"Abandonment" or "Acid rain" or "Acidification" or "Alien species" or "Carbon cycle" or "Clima* change" or "Desertification" or "Exotic species" or "Fragmentation" or "Global warning" or "Habitat change" or "Habitat conversion" or "IAS" or "Intensification" or "Intensive agriculture" or "Invasive species" or "Invasive-alien species" or "Land conversion" or "Land cover and change" or "Land degradation" or "Land use conversion" or "Land-use change" or "Land-use intensity" or "Nitrogen cycle" or "Non-native species" or "Overexploitation" or "Overharvesting" or "Overuse" or "Phosphorous cycle" or "Pollution" or "Sulfure cycle" or "Sulphur cycle" or "Urbanization"

AND

**Functional Diversity**

"Functional richness" or "Functional character*" or "Functional composition" or "Community weight*" or "Functional effect*" or "Functional divergence" or "Functional trait*" or "Dominant trait*" or "Organism providing servic*" or "Functional diversity" or "Service provid* unit" or "Trait effect*" or "Ecological attribute*" or "Ecosystem service provid*" or "Trait range*" or "Trait reponse or response trait" or "Functional group*" or "Effect trait*" or "Functional guild*" or "Functional dispers*" or "Functional redundancy" or "Functional respon*" or “Agregate trait mean” or “Niche complementary” or “growth form*” or “SPU” or “Trait dissimilarit*” or “mass ratio” or “CWM”

AND

**Ecosystem services**

"Ornamental resources" or "Fodder" or "Habitat provision" or "Eco* good*" or "Wild prod*" or "Timber" or "Biofuels" or "Ecosystem* servic*" or "Regulating servic*" or "Nontimber forest" or "Air flow regulation" or "Habitat maintenance" or "Habitat for species" or "Biological control" or "Air quality" or "Provisioning servic*" or "Fiber" or "Erosion control" or "Atmospheric regulation" or "Biocontrol" or "Hazard prevention" or "Bioremediation" or "Carbon sequestration" or "Water quality" or "Fibre" or "Carbon storage" or "Erosion prevent" or "Climate regulation" or "Raw material*" or "Agriculture" or “Livestock” or “Cattle” or "Genetic resources" or "Medicinal resources" or "Disease control" or "Hydrological regulation" or "Invasion resistance" or "Nutrient cycling" or "Pest control" or "Plague control" or "Plague prevention" or "Pollination" or "Sediment retention" or "Seed dispers*" or "Soil fertility" or "Soil formation" or "Soil loss prevention" or "Soil stability" or "Waste treatment" or "Water purification" or "Water regulation" or "Cultural servic*" or "Aesthetic values" or "Cultural heritage" or "Cultural value*" or "Ecological knowledge" or "Environmental education" or "Fishing*" or "Hunting*" or "Identity" or "Inspiration" or "Landscape beauty" or "Recreat*" or "Relax*" or "Sacred places" or "Scientific knowledge" or "Scientific value*" or "Sense of place" or "Spiritual" or "Touris*" or "Tranquility" or "Turis*".
