# Supplementary figures and images for: Trait‐based approaches to analyze links between the drivers of change and ecosystem services: Synthesizing existing evidence and future challenges
Source: Ecol Evol. 2017 Jan 4;7(3):831–44. doi: 10.1002/ece3.2692 (PMC5288245; doi:10.1002/ece3.2692)

**Appendix S2. Scientific literature search flow diagram.**

**
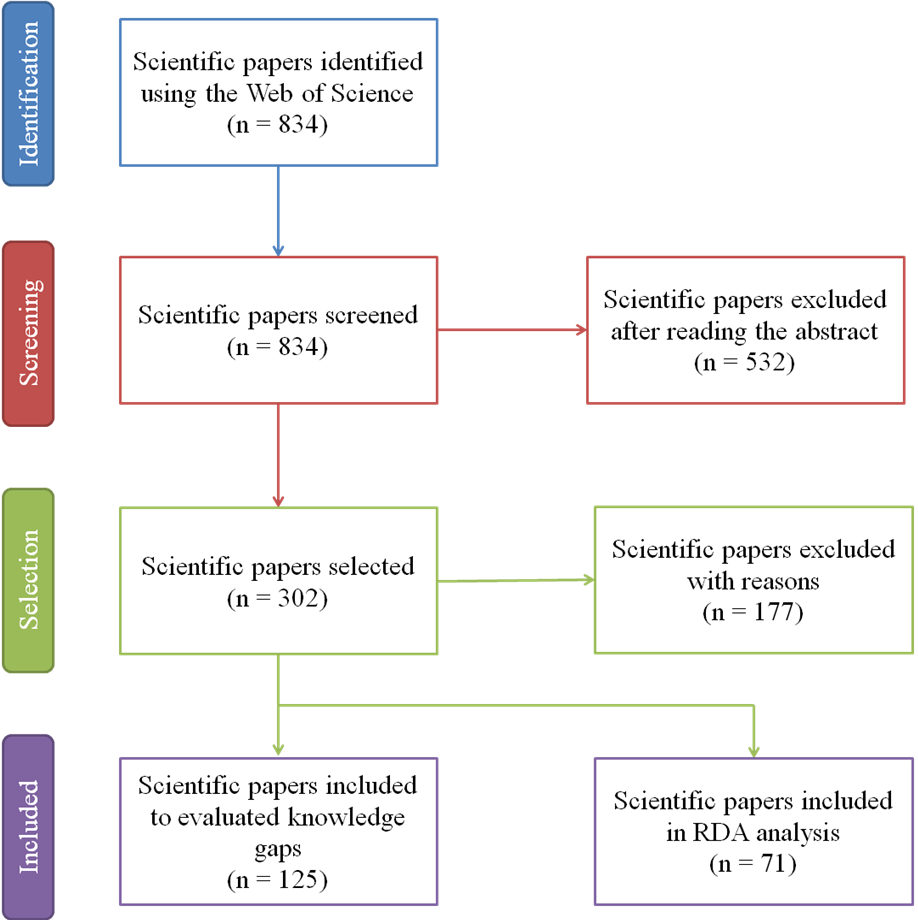
**

Supplement: Supplementary file 2 [file ECE3-7-831-s002.doc]
