# Supplementary material for: Trait‐based approaches to analyze links between the drivers of change and ecosystem services: Synthesizing existing evidence and future challenges
Source: Ecol Evol. 2017 Jan 4;7(3):831–44. doi: 10.1002/ece3.2692 (PMC5288245; doi:10.1002/ece3.2692)
Supplement: Supplementary file 3 [file ECE3-7-831-s003.doc]

**Appendix S3. List of papers considered in the review**

Albrecht, M., Schmid, B., Obrist, M.K., Schüpbach, B., Kleijn, D., Duelli, P., (2010). Effects of ecological compensation meadows on arthropod diversity in adjacent intensively managed grassland. *Biological Conserv*ation, **143**(3), 642-649.

Andresen, E., (2003). Effect of forest fragmentation on dung beetle communities and functional consequences for plant regeneration*. Ecography,* **26**(1), 87-97.

Archaimbault, V., Usseglio‐Polatera, P.H.I.L.I.P.P.E., Garric, J., Wasson, J.G., Babut, M., (2010). Assessing pollution of toxic sediment in streams using bio‐ecological traits of benthic macroinvertebrates. *Freshwater Biology,* **55**(7), 1430-1446.

Atkinson, C.L., Opsahl, S.P., Covich, A.P., Golladay, S.W., Conner, L.M., (2010). Stable isotopic signatures, tissue stoichiometry, and nutrient cycling (C and N) of native and invasive freshwater bivalves*. Journal of the North American Benthological Society,* **29**(2), 496-505.

Avendaño-mendoza, C., Morón-ríos, A., Cano, E.B., León-cortés, J., (2005). Dung beetle community (Coleoptera: Scarabaeidae: Scarabaeinae) in a tropical landscape at the Lachua Region, Guatemala. *Biodiversity and Conservation,* **14**(4), 801-822.

Bardgett, R. D., Mommer, L., De Vries, F. T., (2014). Going underground: root traits as drivers of ecosystem processes. *Trends in Ecology & Evolution*, **29**(12), 692-699.

Barnes, A. D., Emberson, R. M., Krell, F. T., Didham, R. K., (2014). The Role of Species Traits in Mediating Functional Recovery during Matrix Restoration. *PloS One*, **9**(12), e115385.

Barragán, F., Moreno, C.E., Escobar, F., Halffter, G., Navarrete, D., (2011). Negative impacts of human land use on dung beetle functional diversity. *PloS One,* **6**(3), e17976.

Batáry, P., Holzschuh, A., Orci, K.M., Samu, F., Tscharntke, T., (2012). Responses of plant, insect and spider biodiversity to local and landscape scale management intensity in cereal crops and grasslands. *Agriculture Ecosystems & Environment,* **146**(1), 130-136.

Belote, R.T., Jones, R.H., Wieboldt, T.F., (2012). Compositional stability and diversity of vascular plant communities following logging disturbance in Appalachian forests. *Ecological Applications,* **22**(2), 502-516.

Beynon, S.A., Mann, D.J., Slade, E.M., Lewis, O.T., (2012). Species-rich dung beetle communities buffer ecosystem services in perturbed agro‐ecosystems. *Journal of Applied Ecology,* **49**(6), 1365-1372.

Bonet, A., (2004). Secondary succession of semi-arid Mediterranean old-fields in south-eastern Spain: insights for conservation and restoration of degraded lands. *Journal of Arid Environments,* **56**(2), 213-233.

Bregman, T. P., Sekercioglu, C. H., Tobias, J. A., (2014). Global patterns and predictors of bird species responses to forest fragmentation: implications for ecosystem function and conservation. *Biological Conservation*, **169**, 372-383.

Brittain, C., Kremen, C., Klein, A. M., (2013). Biodiversity buffers pollination from changes in environmental conditions. *Global change biology*, **19**(2), 540-547.

Brooks, D.R., Bater, J.E., Clark, S.J., Monteith, D.T., Andrews, C., Corbett, S.J., *et al*., (2012). Large carabid beetle declines in a United Kingdom monitoring network increases evidence for a widespread loss in insect biodiversity. *Journal of Applied Ecology,* **49**(5), 1009-1019.

Brown, K. A., Johnson, S. E., Parks, K. E., Holmes, S. M., Ivoandry, T., Abram, N. K., *et al*., (2013). Use of provisioning ecosystem services drives loss of functional traits across land use intensification gradients in tropical forests in Madagascar. *Biological Conservation*, **161**, 118-127.

Brown, C.S., Rice, K.J., (2010). Effects of belowground resource use complementarity on invasion of constructed grassland plant communities. *Biological Invasions,* **12**(5), 1319-1334.

Busch, D. S., Harvey, C. J., McElhany, P., (2013). Potential impacts of ocean acidification on the Puget Sound food web. ICES Journal of Marine Science: *Journal du Conseil*, **70**(4), 823-833.

Castro, H., Lehsten, V., Lavorel, S., Freitas, H., (2010). Functional response traits in relation to land use change in the Montado. *Agriculture, Ecosystems & Environment,* **137**(1), 183-191.

Cavanaugh, K. C., Gosnell, J. S., Davis, S. L., Ahumada, J., Boundja, P., Clark, D. B., *et al*., (2014). Carbon storage in tropical forests correlates with taxonomic diversity and functional dominance on a global scale. *Global Ecology and Biogeography,* **23**(5), 563-573.

Chillo, V., Anand, M., Ojeda, R.A., (2011). Assessing the use of functional diversity as a measure of ecological resilience in arid rangelands. *Ecosystems,* **14**(7), 1168-1177.

Chillo, V., Ojeda, R., (2014). Disentangling ecosystem responses to livestock grazing in drylands. *Agriculture, Ecosystems & Environment*, **197**, 271-277.

Cinner, J.E., McClanahan, T.R., Graham, N.A., Pratchett, M.S., Wilson, S.K., Raina, J.B., (2009). Gear‐based fisheries management as a potential adaptive response to climate change and coral mortality. *Journal of Applied Ecology,* **46**(3), 724-732.

Clough, Y., Dwi Putra, D., Pitopang, R., Tscharntke, T., (2009). Local and landscape factors determine functional bird diversity in Indonesian cacao agroforestry. *Biological Conservation,* **142**(5), 1032-1041.

Cole, L.J., Brocklehurst, S., Elston, D.A., McCracken, D.I., (2012). Riparian field margins: can they enhance the functional structure of ground beetle (Coleoptera: Carabidae) assemblages in intensively managed grassland landscapes? *Journal of Applied Ecology,* **49**(6), 1384-1395.

Cornelissen, J. H., Makoto, K., (2014). Winter climate change, plant traits and nutrient and carbon cycling in cold biomes. *Ecological research*, **29**(4), 517-527.

Cuffney, T.F., Brightbill, R.A., May, J.T., Waite, I.R., (2010). Responses of benthic macroinvertebrates to environmental changes associated with urbanization in nine metropolitan areas. *Ecological Applications,* **20**(5), 1384-1401.

da Silva Dias, N., Zanetti, R., Santos, M. S., Peñaflor, M. F. G. V., Broglio, S. M. F., Delabie, J. H. C., (2013). The impact of coffee and pasture agriculture on predatory and omnivorous leaf-litter ants. *Journal of Insect Science*, **13**(1), 29.

Dahms, H., Mayr, S., Birkhofer, K., Chauvat, M., Melnichnova, E., Wolters, V., *et al*., (2010). Contrasting diversity patterns of epigeic arthropods between grasslands of high and low agronomic potential. *Basic and Applied Ecology,* **11**(1), 6-14.

Dai, J., Wang, H., Ge, Q., (2013). Multiple phenological responses to climate change among 42 plant species in Xi’an, China. *International Journal of Biometeorology*, **57**(5), 749-758.

de Villalobos, A.E., Zalba, S.M., (2010). Continuous feral horse grazing and grazing exclusion in mountain pampean grasslands in Argentina. *Acta Oecologica,* **36**(5), 514-519.

Deikumah, J. P., McAlpine, C. A., Maron, M., (2013). Matrix intensification alters avian functional group composition in adjacent rainforest fragments. *PloS One,* e74852.

Dukes, J.S., (2001). Biodiversity and invasibility in grassland microcosms. *Oecologia,* **126**(4), 563-568.

Edwards, F. A., Edwards, D. P., Larsen, T. H., Hsu, W. W., Benedick, S., Chung, A., *et al.*., (2014). Does logging and forest conversion to oil palm agriculture alter functional diversity in a biodiversity hotspot? *Animal Conservation*, **17**(2), 163-173.

Escribano‐Avila, G., Calviño‐Cancela, M., Pías, B., Virgós, E., Valladares, F., Escudero, A., (2014). Diverse guilds provide complementary dispersal services in a woodland expansion process after land abandonment. *Journal of Applied Ecology*, **51**(6), 1701-1711.

Ferger, S. W., Böhning-Gaese, K., Wilcke, W., Oelmann, Y., Schleuning, M., (2013). Distinct carbon sources indicate strong differentiation between tropical forest and farmland bird communities. *Oecologia*, **171**(2), 473-486.

Fernández-Lugo, S., Bermejo, L. A., de Nascimento, L., Méndez, J., Naranjo-Cigala, A., & Arévalo, J. R., (2013). Productivity: key factor affecting grazing exclusion effects on vegetation and soil. *Plant ecology*, **214**(4), 641-656.

Fontana, V., Radtke, A., Walde, J., Tasser, E., Wilhalm, T., Zerbe, S., *et al*., (2014). What plant traits tell us: Consequences of land-use change of a traditional agro-forest system on biodiversity and ecosystem service provision. *Agriculture, Ecosystems & Environment*, **186**, 44-53.

Franklin, K., (2012). The remarkable resilience of ant assemblages following major vegetation change in an arid ecosystem. *Biological Conservation,* **148**(1), 96-105.

Gabriel, D., Roschewitz, I., Tscharntke, T., Thies, C., (2006). Beta diversity at different spatial scales: plant communities in organic and conventional agriculture. *Ecological Applications,* **16**(5), 2011-2021.

García, D., Martínez, D., Herrera, J. M., Morales, J. M., (2013). Functional heterogeneity in a plant–frugivore assemblage enhances seed dispersal resilience to habitat loss. *Ecography*, **36**(2), 197-208.

Gard, B., Bretagnolle, F., Dessaint, F., Laitung, B., (2013). Invasive and native populations of common ragweed exhibit strong tolerance to foliar damage. *Basic and Applied Ecology*, **14**(1), 28-35.

Grass, I., Berens, D. G., Farwig, N., (2014). Natural habitat loss and exotic plants reduce the functional diversity of flower visitors in a heterogeneous subtropical landscape. *Functional Ecology*, **28**(5), 1117-1126.

Gray, C. L., Slade, E. M., Mann, D. J., Lewis, O. T., (2014). Do riparian reserves support dung beetle biodiversity and ecosystem services in oil palm‐dominated tropical landscapes? *Ecology and Evolution*, **4**(7), 1049-1060.

Griffith, G.P., Fulton, E.A., Richardson, A.J., (2011). Effects of fishing and acidification‐related benthic mortality on the southeast Australian marine ecosystem. *Global Change Biology,* **17**(10), 3058-3074.

Griffith, G.P., Fulton, E.A., Gorton, R., Richardson, A.J., (2012). Predicting Interactions among Fishing, Ocean Warming, and Ocean Acidification in a Marine System with Whole-Ecosystem Models. *Conservation Biology,* **26**(6), 1145-1152.

Griffiths, S.P., Young, J.W., Lansdell, M.J., Campbell, R.A., Hampton, J., Hoyle, S.D., *et al.*, (2010). Ecological effects of longline fishing and climate change on the pelagic ecosystem off eastern Australia. *Reviews in Fish Biology,* **20**(2), 239-272.

Guerrero, I., Morales, M.B., Oñate, J.J., Aavik, T., Bengtsson, J., Berendse, F., *et al*., (2011). Taxonomic and functional diversity of farmland bird communities across Europe: effects of biogeography and agricultural intensification. *Biodiversity and Conservation,* **20**(14), 3663-3681.

Häger, A., (2012). The effects of management and plant diversity on carbon storage in coffee agroforestry systems in Costa Rica. *Agroforestry Systems,* **86**(2), 159-174.

Hajian-Forooshani, Z., Gonthier, D. J., Marín, L., Iverson, A. L., Perfecto, I., (2014). Changes in species diversity of arboreal spiders in Mexican coffee agroecosystems: untangling the web of local and landscape influences driving diversity*. PeerJ*, **2**, e623.

Har-Edom, O.L., Sternberg, M., (2010). Invasive species and climate change: Conyza canadensis (L.) Cronquist as a tool for assessing the invasibility of natural plant communities along an aridity gradient. *Biological Invasions,* **12**(7), 1953-1960.

Hoehn, P., Tscharntke, T., Tylianakis, J.M., Steffan-Dewenter, I., (2008). Functional group diversity of bee pollinators increases crop yield. *Proceedings of the Royal Society B: Biological Sciences,* **275**(1648), 2283-2291.

Holzschuh, A., Steffan‐Dewenter, I., Tscharntke, T., (2010). How do landscape composition and configuration, organic farming and fallow strips affect the diversity of bees, wasps and their parasitoids? *Journal of Animal Ecology,* **79**(2), 491-500.

House, A.P., Burwell, C.J., Brown, S.D., Walters, B.J., (2012). Agricultural matrix provides modest habitat value for ants on mixed farms in eastern Australia. *Journal of Insect Conservation,* **16**(1), 1-12.

Jansson, Å., Polasky, S., (2010). Quantifying biodiversity for building resilience for food security in urban landscapes: Getting down to business*. Ecology and Society,* **15**(3), 20.

Jiguet, F., Devictor, V., Julliard, R., Couvet, D., (2012). French citizens monitoring ordinary birds provide tools for conservation and ecological sciences. *Acta Oecologica,* **44**, 58-66.

Jones, W.M., Fraser, L.H., Curtis, P.J., (2011). Plant community functional shifts in response to livestock grazing in intermountain depressional wetlands in British Columbia, Canada. *Biological Conservation,* **144**(1), 511-517.

José-María, L., Blanco-Moreno, J.M., Armengot, L., Sans, F.X., (2011). How does agricultural intensification modulate changes in plant community composition? *Agriculture, Ecosystems & Environment,* **145**(1), 77-84.

Kagezi, G.H., Kaib, M., Nyeko, P., Bakuneeta, C., Schädler, M., Brandl, R., (2011). Decomposition of tissue baits and termite density along a gradient of human land‐use intensification in Western Kenya. *African Journal of Ecology,* **49**(3), 267-276.

Kang, W., Hoffmeister, M., Martin, E. A., Steffan-Dewenter, I., Han, D., Lee, D., (2013). Effects of management and structural connectivity on the plant communities of organic vegetable field margins in South Korea. *Ecological Research*, **28**(6), 991-1002.

Knapp, S., Dinsmore, L., Fissore, C., Hobbie, S.E., Jakobsdottir, I., Kattge, J., *et al*., (2012). Phylogenetic and functional characteristics of household yard floras and their changes along an urbanization gradient. *Ecology,* **93**(sp8), S83-S98.

Kooyman, R. M., Zanne, A. E., Gallagher, R. V., Cornwell, W., Rossetto, M., O'Connor, P., *et al*., (2013). Effects of growth form and functional traits on response of woody plants to clearing and fragmentation of subtropical rainforest. *Conservation Biology*, **27**(6), 1468-1477.

Koyanagi, T., Kusumoto, Y., Yamamoto, S., Okubo, S., Iwasaki, N., Takeuchi, K., (2012). Grassland plant functional groups exhibit distinct time-lags in response to historical landscape change. *Plant Ecology,* **213**(2), 327-338.

Krause, B., Culmsee, H., (2013). The significance of habitat continuity and current management on the compositional and functional diversity of grasslands in the uplands of Lower Saxony, Germany. *Flora-Morphology, Distribution, Functional Ecology of Plants*, **208**(5), 299-311.

Kudavidanage, E.P., Qie, L., Lee, J.S.H., (2012). Linking biodiversity and ecosystem functioning of dung beetles in South and Southeast Asian tropical rainforests. The *Raffles bulletin of zoology,* (25), 141-154.

Kühner, A., Kleyer, M., (2008). A parsimonious combination of functional traits predicting plant response to disturbance and soil fertility. *Journal of Vegetation Science,* **19**(5), 681-692.

Kyle, G., Leishman, M.R., (2009). Functional trait differences between extant exotic, native and extinct native plants in the Hunter River, NSW: a potential tool in riparian rehabilitation. *River Research and Applications,* **25**(7), 892-903.

Laliberté, E., Tylianakis, J.M., (2012). Cascading effects of long-term land-use changes on plant traits and ecosystem functioning. *Ecology,* **93**(1), 145-155.

Lavorel, S., Grigulis, K., Lamarque, P., Colace, M.P., Garden, D., Girel, J., *et al.*, (2011). Using plant functional traits to understand the landscape distribution of multiple ecosystem services. *Journal of Ecology,* **99**(1), 135-147.

Lavorel, S., Storkey, J., Bardgett, R. D., Bello, F., Berg, M. P., Roux, X., *et al*., (2013). A novel framework for linking functional diversity of plants with other trophic levels for the quantification of ecosystem services. *Journal of Vegetation Science*, **24**(5), 942-948.

Li, F. R., Liu, J. L., Sun, T. S., Jin, B. W., Chen, L. J., (2014). Converting natural vegetation to farmland alters functional structure of ground-dwelling beetles and spiders in a desert oasis. *Journal of Insect Conservation*, **18**(1), 57-67.

Lin, B.B., Flynn, D.F., Bunker, D.E., Uriarte, M., Naeem, S., (2011). The effect of agricultural diversity and crop choice on functional capacity change in grassland conversions. *Journal of Applied Ecology,* **48**(3), 609-618.

Lindsay, E.A., Cunningham, S.A., (2009). Livestock grazing exclusion and microhabitat variation affect invertebrates and litter decomposition rates in woodland remnants. *Forest Ecology and Management,* **258**(2), 178-187.

Liu, Y., Rothenwöhrer, C., Scherber, C., Batáry, P., Elek, Z., Steckel, J., *et al*., (2014). Functional beetle diversity in managed grasslands: effects of region, landscape context and land use intensity. *Landscape Ecology*, **29**(3), 529-540.

Livingston, G., Jha, S., Vega, A., Gilbert, L., (2013). Conservation value and permeability of neotropical oil palm landscapes for orchid bees. *PloS One*, **8** (10), e78523.

Llop, E., Pinho, P., Matos, P., Pereira, M.J., Branquinho, C., (2012). The use of lichen functional groups as indicators of air quality in a Mediterranean urban environment. *Ecological Indicators,* **13**(1), 215-221.

Lopes, A.V., Girão, L.C., Santos, B.A., Peres, C.A., Tabarelli, M., (2009). Long-term erosion of tree reproductive trait diversity in edge-dominated Atlantic forest fragments. *Biological Conservation,* **142**(6), 1154-1165.

Luck, G. W., Carter, A., Smallbone, L., (2013). Changes in bird functional diversity across multiple land uses: interpretations of functional redundancy depend on functional group identity. *Plos One,* e63671.

Ma, M., Herzon, I., (2014). Plant functional diversity in agricultural margins and fallow fields varies with landscape complexity level: Conservation implications. *Journal for Nature Conservation*, **22**(6), 525-531.

Maron, J., Marler, M., (2007). Native plant diversity resists invasion at both low and high resource levels. *Ecology,* **88**(10), 2651-2661.

Mayfield, M.M., Bonser, S.P., Morgan, J.W., Aubin, I., McNamara, S., Vesk, P.A., (2010). What does species richness tell us about functional trait diversity? Predictions and evidence for responses of species and functional trait diversity to land‐use change. *Global Ecology and Biogeography,* **19**(4), 423-431.

McIntyre, S., (2008). The role of plant leaf attributes in linking land use to ecosystem function in temperate grassy vegetation. *Agriculture, Ecosystems & Environment,* **128**(4), 251-258.

McKie, B.G., Malmqvist, B., (2009). Assessing ecosystem functioning in streams affected by forest management: increased leaf decomposition occurs without changes to the composition of benthic assemblages. *Freshwater Biology,* **54**(10), 2086-2100.

Mouysset, L., Doyen, L., Jiguet, F., (2012). Different policy scenarios to promote various targets of biodiversity. *Ecological Indicators,* **14**(1), 209-221.

Newbold, T., Scharlemann, J. P., Butchart, S. H., Şekercioğlu, Ç. H., Alkemade, R., Booth, H., *et al*., (2013). Ecological traits affect the response of tropical forest bird species to land-use intensity. *Proceedings of the Royal Society of London B: Biological Sciences*, **280**(1750), 20122131.

Newbold, T., Scharlemann, J. P., Butchart, S. H., Şekercioğlu, Ç. H., Joppa, L., Alkemade, R., *et al*., (2014). Functional traits, land‐use change and the structure of present and future bird communities in tropical forests. *Global Ecology and Biogeography*, **23**(10), 1073-1084.

Nhiwatiwa, T., De Bie, T., Vervaeke, B., Barson, M., Stevens, M., Vanhove, M.P., *et al*., (2009). Invertebrate communities in dry-season pools of a large subtropical river: patterns and processes. *Hydrobiologia,* **630**(1), 169-186.

Norfolk, O., Eichhorn, M. P., Gilbert, F., (2013). Traditional agricultural gardens conserve wild plants and functional richness in arid South Sinai*. Basic and Applied Ecology*, **14**(8), 659-669.

Osuri, A. M., Kumar, V. S., Sankaran, M., (2014). Altered stand structure and tree allometry reduce carbon storage in evergreen forest fragments in India’s Western Ghats. *Forest Ecology and Management*, **329**, 375-383.

Pakeman, R. J., (2014). Leaf Dry Matter Content Predicts Herbivore Productivity, but Its Functional Diversity Is Positively Related to Resilience in Grasslands. *PloS One,* e101876.

Pauli, N., Barrios, E., Conacher, A.J., Oberthür, T., (2011). Soil macrofauna in agricultural landscapes dominated by the Quesungual Slash-and-Mulch Agroforestry System, western Honduras. *Applied Soil Ecology,* **47**(2), 119-132.

Pauw, A., Louw, K., (2012). Urbanization drives a reduction in functional diversity in a guild of nectar-feeding birds. *Ecology and Society,* **17**(2), 27.

Penone, C., Machon, N., Julliard, R., Le Viol, I., (2012). Do railway edges provide functional connectivity for plant communities in an urban context? *Biological Conservation,* **148**(1), 126-133.

Perelman, S.B., Chaneton, E.J., Batista, W.B., Burkart, S.E., Leon, R.J., (2007). Habitat stress, species pool size and biotic resistance influence exotic plant richness in the Flooding Pampa grasslands. *Journal of Ecology,* **95**(4), 662-673.

Perry, R.I., Cury, P., Brander, K., Jennings, S., Möllmann, C., Planque, B., (2010). Sensitivity of marine systems to climate and fishing: concepts, issues and management responses. *Journal of Marine Systems,* **79**(3), 427-435.

Pinho, P., Bergamini, A., Carvalho, P., Branquinho, C., Stofer, S., Scheidegger, C., *et al*., (2012). Lichen functional groups as ecological indicators of the effects of land-use in Mediterranean ecosystems. *Ecological Indicators,* **15**(1), 36-42.

Pyšek, P., Jarošík, V., Chytrý, M., Danihelka, J., Kühn, I., Pergl, J., *et al*., (2011). Successful invaders co-opt pollinators of native flora and accumulate insect pollinators with increasing residence time. *Ecological Monographs,* **81**(2), 277-293.

Qian, S.S., Cuffney, T.F., McMahon, G., (2012). Multinomial regression for analyzing macroinvertebrate assemblage composition data. *Freshwater Science,* **31**(3), 681-694.

Rader, R., Bartomeus, I., Tylianakis, J. M., Laliberté, E., (2014). The winners and losers of land use intensification: pollinator community disassembly is non‐random and alters functional diversity. *Diversity and Distributions*, **20**(8), 908-917.

Robertson, B.A., Porter, C., Landis, D.A., Schemske, D.W., (2012). Agroenergy crops influence the diversity, biomass, and guild structure of terrestrial arthropod communities. *Bioenergy Research,* **5**(1), 179-188.

Rosenlew, H., Roslin, T., (2008). Habitat fragmentation and the functional efficiency of temperate dung beetles. *Oikos,* **117**(11), 1659-1666.

Rusch, A., Birkhofer, K., Bommarco, R., Smith, H. G., Ekbom, B., (2014). Management intensity at field and landscape levels affects the structure of generalist predator communities. *Oecologia*, **175**(3), 971-983.

Rusch, A., Bommarco, R., Chiverton, P., Öberg, S., Wallin, H., Wiktelius, S., *et al*, (2013). Response of ground beetle (Coleoptera, Carabidae) communities to changes in agricultural policies in Sweden over two decades. *Agriculture, Ecosystems & Environment*, **176**, 63-69.

Rzanny, M., Voigt, W., (2012). Complexity of multitrophic interactions in a grassland ecosystem depends on plant species diversity. *Journal of Applied Ecology,* **81**(3), 614-627.

Scharfy, D., Eggenschwiler, H., Olde Venterink, H., Edwards, P.J., Güsewell, S., (2009). The invasive alien plant species Solidago gigantea alters ecosystem properties across habitats with differing fertility. *Journal of Vegetation Science,* **20**(6), 1072-1085.

Schleicher, A., Biedermann, R., Kleyer, M., (2011). Dispersal traits determine plant response to habitat connectivity in an urban landscape. *Landscape Ecology,* **26**(4), 529-540.

Schmera, D., Baur, B., Erős, T., (2012). Does functional redundancy of communities provide insurance against human disturbances? An analysis using regional-scale stream invertebrate data. *Hydrobiologia,* **693**(1), 183-194.

Sheffield, C. S., Kevan, P. G., Pindar, A., Packer, L., (2013). Bee (Hymenoptera: Apoidea) diversity within apple orchards and old fields in the Annapolis Valley, Nova Scotia, Canada. *The Canadian Entomologist*, **145**(01), 94-114.

Soudzilovskaia, N. A., Elumeeva, T. G., Onipchenko, V. G., Shidakov, I. I., Salpagarova, F. S., Khubiev, A. B., *et al*., (2013). Functional traits predict relationship between plant abundance dynamic and long-term climate warming. *Proceedings of the National Academy of Sciences*, **110**(45), 18180-18184.

Steckel, J., Westphal, C., Peters, M. K., Bellach, M., Rothenwoehrer, C., Erasmi, S., *et al*., (2014). Landscape composition and configuration differently affect trap-nesting bees, wasps and their antagonists. *Biological Conservation*, **172**, 56-64.

Storkey, J., Brooks, D., Haughton, A., Hawes, C., Smith, B. M., Holland, J. M., (2013). Using functional traits to quantify the value of plant communities to invertebrate ecosystem service providers in arable landscapes. *Journal of ecology*, **101**(1), 38-46.

Tadesse, G., Zavaleta, E., Shennan, C., (2014). Effects of land-use changes on woody species distribution and above-ground carbon storage of forest-coffee systems. *Agriculture, Ecosystems & Environment*, **197**, 21-30.

Thyresson, M., Crona, B., Nyström, M., de la Torre-Castro, M., Jiddawi, N., (2013). Tracing value chains to understand effects of trade on coral reef fish in Zanzibar, Tanzania. *Marine Policy*, **38**, 246-256.

Tocco, C., Probo, M., Lonati, M., Lombardi, G., Negro, M., Nervo, B., *et al*., (2013). Pastoral practices to reverse shrub encroachment of sub-alpine grasslands: dung beetles (Coleoptera, Scarabaeoidea) respond more quickly than vegetation. *PloS One,* e83344.

Trivellone, V., Paltrinieri, L.P., Jermini, M., Moretti, M., (2012). Management pressure drives leafhopper communities in vineyards in Southern Switzerland. *Insect Conservation and Diversity,* **5**(1), 75-85.

Vauramo, S., Setälä, H., (2010). Urban belowground food-web responses to plant community manipulation–Impacts on nutrient dynamics. *Landscape Urban Planning,* **97**(1), 1-10.

Verón, S.R., Paruelo, J.M., Oesterheld, M., (2011). Grazing-induced losses of biodiversity affect the transpiration of an arid ecosystem. *Oecologia,* **165**(2), 501-510.

Weiss, L., Pfestorf, H., May, F., Körner, K., Boch, S., Fischer, M., *et al*., (2014). Grazing response patterns indicate isolation of semi‐natural European grasslands. *Oikos*, **123**(5), 599-612.

Whitfeld, T. J., Roth, A. M., Lodge, A. G., Eisenhauer, N., Frelich, L. E., Reich, P. B., (2014). Resident plant diversity and introduced earthworms have contrasting effects on the success of invasive plants. *Biological Invasions*, **16**(10), 2181-2193.

Williams, N.M., Crone, E.E., Minckley, R.L., Packer, L., Potts, S.G., (2010). Ecological and life-history traits predict bee species responses to environmental disturbances. *Biological Conservation,* **143**(10), 2280-2291.

Wilson, S.K., Fisher, R., Pratchett, M.S., Graham, N.A.J., Dulvy, N.K., Turner, R.A., *et al*., (2008). Exploitation and habitat degradation as agents of change within coral reef fish communities. *Global Change Biology,* **14**(12), 2796-2809.

Winqvist, C., Bengtsson, J., Öckinger, E., Aavik, T., Berendse, F., Clement, L. W., *et al*., (2014). Species’ traits influence ground beetle responses to farm and landscape level agricultural intensification in Europe. *Journal of Insect Conservation,* **18**(5), 837-846.

Zhao, D., Li, F., Yang, Q., Wang, R., Song, Y., Tao, Y., (2013). The influence of different types of urban land use on soil microbial biomass and functional diversity in Beijing, China*. Soil Use and Management*, **29**(2), 230-239.

Ziter, C., Bennett, E. M., Gonzalez, A., (2013). Functional diversity and management mediate aboveground carbon stocks in small forest fragments. *Ecosphere*, **4**(7), art85.
