# Supplementary material for: Trait‐based approaches to analyze links between the drivers of change and ecosystem services: Synthesizing existing evidence and future challenges
Source: Ecol Evol. 2017 Jan 4;7(3):831–44. doi: 10.1002/ece3.2692 (PMC5288245; doi:10.1002/ece3.2692)
Supplement: Supplementary file 4 [file ECE3-7-831-s004.doc]

**Appendix S4**. List of variables considered and the corresponding attributes.

| Variables | Attributes |
| --- | --- |
| *Publication characteristics* |  |
| Number of authors |  |
| Category of Web of Science | Ecology, Botany, Biodiversity and Conservation, Hydrobiology, Zoology, Environmental Sciences, Others |
| Interdisciplinary team | Yes, No |
| Year of publication |  |
| Journal |  |
| Research area |  |
| Type of study | Experimental, Observational, Predictive |
|  |  |
| *Location* |  |
| Location of the study area |  |
| *Methodological approach* |  |
| System border definition | Administrative, Biophysical |
| Spatial scale | Global, Regional, National, Subnational, Local |
| Taxonomic group | Invertebrates, Vegetation, Vertebrates |
| Period of study | Monitoring, Punctual |
| *Characteristics of ecosystems* |  |
| Type of ecosystem (based on Millennium Ecosystem Assessment) | Coastal, Cultivated, Dryland, Forest, Inland water, Marine, Polar, Urban, Interaction |
| Protected area | Yes, No |
| *Direct Drivers of change* |  |
| Type of Direct Drivers of change | Pollution, Alien species, Climate change, Land use change, Overexploitation |
| *Functional Traits* |  |
| Functional traits used in the study |  |
| *Ecosystem services* |  |
| Type of ecosystem services | Cultural, Provisioning, Regulating |
| *Statistical relation* |  |
| Driver-Functional diversity | Positive, Negative, Not significant |
| Functional diversity-Ecosystem services | Positive, Negative, Not significant |
| Driver-Ecosystem services | Positive, Negative, Not significant |
