# Supplementary material for: Trait‐based approaches to analyze links between the drivers of change and ecosystem services: Synthesizing existing evidence and future challenges
Source: Ecol Evol. 2017 Jan 4;7(3):831–44. doi: 10.1002/ece3.2692 (PMC5288245; doi:10.1002/ece3.2692)
Supplement: Supplementary file 5 [file ECE3-7-831-s005.doc]

**Appendix S5. List of the functional traits for each taxonomic group recorded in our literature sampling. The last column shows the number of studies which used each functional trait.**

| **Functional trait** | **Taxonomic group** | | | | | **Nº of studies** |
| --- | --- | --- | --- | --- | --- | --- |
|  | Vegetation | Invertebrates | | | Vertebrates |  |
| Allometries |  | |  |  | | 1 |
| Bark thickness |  | |  |  | | 1 |
| Bill length |  | |  |  | | 1 |
| Breeding season |  | |  |  | | 2 |
| C:N ratio |  | |  |  | | 1 |
| Canopy structure |  | |  |  | | 2 |
| Carbon content |  | |  |  | | 2 |
| Clutch size |  | |  |  | | 1 |
| Cold tolerance |  | |  |  | | 1 |
| Consumption |  | |  |  | | 2 |
| Diameter at breast height |  | |  |  | |  |
| Diel activity |  | |  |  | | 5 |
| Diet |  | |  |  | | 24 |
| Dispersal activity |  | |  |  | | 20 |
| Dry matter content |  | |  |  | | 4 |
| Duration of flower visitation |  | |  |  | | 1 |
| Feeding habit |  | |  |  | | 16 |
| Flocking behaviour |  | |  |  | | 1 |
| Flower color |  | |  |  | | 1 |
| Foraging |  | |  |  | | 6 |
| Generation length |  | |  |  | | 2 |
| Growth form |  | |  |  | | 20 |
| Growth rate |  | |  |  | | 2 |
| Habitat dependency |  | |  |  | | 21 |
| Leaf morphology |  | |  |  | | 6 |
| Leaf physiology |  | |  |  | | 1 |
| Leaf water content |  | |  |  | | 1 |
| Life cycle |  | |  |  | | 13 |
| Litter abundance |  | |  |  | | 5 |
| Mating strategy |  | |  |  | | 1 |
| Maximum canopy height |  | |  |  | | 7 |
| Method of dung removal |  | |  |  | | 1 |
| Microclimate moisture preference |  | |  |  | | 2 |
| Migration |  | |  |  | | 3 |
| Mobility |  | |  |  | | 3 |
| Nectar and pollen production |  | |  |  | | 1 |
| Nesting |  | |  |  | | 3 |
| Nitrogen content |  | |  |  | | 3 |
| Nitrogen fixing |  | |  |  | | 5 |
| Nº of visited flowers |  | |  |  | | 1 |
| Number of cotyledons |  | |  |  | | 1 |
| Nutrient mineralisation |  | |  |  | | 1 |
| Onset and/or duration of flowering |  | |  |  | | 3 |
| Overwintering stage |  | |  |  | | 3 |
| Parasitism |  | | | | | 3 |
| Phenology |  | |  |  | | 2 |
| Photosynthesis traits |  | |  |  | | 2 |
| Pollen distribution |  | |  |  | | 1 |
| Pollinating |  | |  |  | | 5 |
| Proboscis morphology |  | |  |  | | 2 |
| Production |  | |  |  | | 3 |
| Pronotum width |  | |  |  | | 1 |
| Recruitment |  | |  |  | | 1 |
| Reproductive effort |  | |  |  | | 3 |
| Reproductive success |  | |  |  | | 1 |
| Root morphology |  | |  |  | | 2 |
| Root physiology |  | |  |  | | 1 |
| Root:shoot ratio |  | |  |  | | 1 |
| Seed mass |  | |  |  | | 10 |
| Seed number |  | |  |  | | 1 |
| Self-compatibily |  | |  |  | | 1 |
| Shade tolerance |  | |  |  | | 1 |
| Size |  | |  |  | | 37 |
| Social organisation |  | |  |  | | 2 |
| Sociality |  | |  |  | | 1 |
| Specific leaf area |  | |  |  | | 16 |
| Storage organs |  | |  |  | | 1 |
| Tongue length |  | |  |  | | 1 |
| Torpor |  | |  |  | | 1 |
| Trophic level |  | |  |  | | 12 |
| Vegetative reproduction |  | |  |  | | 3 |
| Vertical distribution |  | |  |  | | 1 |
| Wing morphology |  | |  |  | | 1 |
| Wood density |  | |  |  | | 3 |
| Woodiness |  | |  |  | | 4 |
