# Supplementary material for: Trait‐based approaches to analyze links between the drivers of change and ecosystem services: Synthesizing existing evidence and future challenges
Source: Ecol Evol. 2017 Jan 4;7(3):831–44. doi: 10.1002/ece3.2692 (PMC5288245; doi:10.1002/ece3.2692)
Supplement: Supplementary file 6 [file ECE3-7-831-s006.doc]

**Appendix S6**. **Scores for the redundancy analysis (RDA) variables and statistics. Bold values represent those response traits (dependent variables) and those explanatory variables with scores > 0.2**

- Vegetation group

|  | Axis1 | Axis2 | Axis3 |
| --- | --- | --- | --- |
| *Dependent variables (response traits)* |  |  |  |
| Nitrogen fixing | -0,041 | -0,025 | 0,008 |
| Size | -0,078 | **0,312** | -0,035 |
| Dispersal activity | -0,160 | **0,261** | -0,019 |
| Life cycle | **0,415** | -0,041 | 0,032 |
| Seed mass | -0,144 | -0,088 | 0,028 |
| Specific leaf area | **-0,247** | -0,152 | 0,048 |
| Parasitism | **0,203** | 0,044 | 0,272 |
| Pollinating | -0,021 | -0,013 | 0,004 |
| Leaf morphology | -0,123 | -0,076 | 0,024 |
| Growth form | 0,191 | -0,098 | -0,236 |
| Maximum canopy height | -0,144 | -0,088 | 0,028 |
| Woodiness | -0,041 | -0,025 | 0,008 |
| Vegetative reproduction | -0,041 | -0,025 | 0,008 |
| Growth rate | -0,041 | -0,025 | 0,008 |
| Nitrogen content | -0,041 | -0,025 | 0,008 |
| Wood density | -0,103 | -0,063 | 0,020 |
| Bark thickness | -0,082 | -0,051 | 0,016 |
| Leaf water content | -0,082 | -0,051 | 0,016 |
| Diameter at breast high | -0,021 | -0,013 | 0,004 |
| *Explanatory variables (direct drivers)* |  |  |  |
| Alien species | **0,398** | 0,053 | 0,090 |
| Climate change | -0,056 | **0,515** | -0,063 |
| Land use change | **-0,318** | -0,258 | 0,300 |
| Overexploitation | **0,308** | -0,044 | -0,258 |
| Eigenvalue | 0,620 | 0,380 | 0,204 |
| Percentage variance explained | 33,451 | 20,490 | 11,022 |
| Cumulative % variance explained | 33,451 | 53,941 | 64,963 |
| Total inertia | 30,110 | 18,443 | 9,921 |

- Invertebrates group

|  | Axis1 |
| --- | --- |
| *Dependent variables (response traits)* |  |
| Size | 0,000 |
| Feeding habit | -0,126 |
| Diel activity | **0,252** |
| Dispersal activity | -0,063 |
| Mobility | -0,021 |
| Foraging | -0,042 |
| Diet | -0,063 |
| Nesting | -0,021 |
| Sociality | -0,021 |
| Pollinating | **0,273** |
| Pronotum width | -0,021 |
| Method of dung removal | -0,021 |
| *Explanatory variables (direct drivers)* |  |
| Climate change | **0,622** |
| Land use change | **-0,622** |
| Eigenvalue | 0,031 |
| Percentage variance explained | 100,000 |
| Cumulative % variance explained | 100,000 |
| Total inertia | 3,091 |

- **Vertebrates group**

|  | Axis1 |
| --- | --- |
| *Dependent variables (response traits)* |  |
| Size | **-0,205** |
| Feeding habit | **0,478** |
| Foraging | -0,137 |
| Diet | 0,102 |
| Trophic level | -0,068 |
| Habitat dependency | -0,102 |
| Wing morphology | -0,034 |
| Torpor | -0,068 |
| *Explanatory variables (direct drivers)* |  |
| Land use change | **-0,841** |
| Overexploitation | **0,841** |
| Eigenvalue | 0,094 |
| Percentage variance explained | 100,000 |
| Cumulative % variance explained | 100,000 |
| Total inertia | 7,857 |
