# Supplementary material for: Trait‐based approaches to analyze links between the drivers of change and ecosystem services: Synthesizing existing evidence and future challenges
Source: Ecol Evol. 2017 Jan 4;7(3):831–44. doi: 10.1002/ece3.2692 (PMC5288245; doi:10.1002/ece3.2692)
Supplement: Supplementary file 7 [file ECE3-7-831-s007.doc]

**Appendix S7**. **Scores for the redundancy analysis variables and statistics. Bold values represent those ecosystem services (dependent variables) and those explanatory variables with scores > 0.2**

- Vegetation group

|  | Axis1 | Axis2 | Axis3 |
| --- | --- | --- | --- |
| *Dependent variables (ecosystem services)* |  |  |  |
| Invasion resistance | -0,094 | **-0,268** | -0,081 |
| Pollination | -0,167 | **-0,356** | 0,354 |
| Nutrient cycling | **0,840** | **0,213** | -0,150 |
| Seed dispersion | 0,011 | -0,103 | -0,283 |
| Soil fertility | **0,529** | 0,181 | 0,388 |
| Carbon cycling | **-0,495** | **0,723** | 0,050 |
| *Explanatory variables (effect traits)* |  |  |  |
| Litter abundance | **-0,248** | **0,365** | 0,022 |
| Nitrogen fixing | 0,141 | -0,003 | -0,163 |
| Size | **0,256** | 0,061 | -0,194 |
| Dispersal activity | -0,012 | **-0,304** | -0,162 |
| Life cycle | **0,590** | 0,001 | 0,297 |
| Seed mass | 0,025 | 0,004 | -0,535 |
| Specific leaf area | 0,145 | 0,083 | -0,259 |
| Pollinating | -0,124 | **-0,277** | 0,349 |
| Leaf morphology | **0,222** | **0,234** | 0,173 |
| Growth form | **0,642** | 0,009 | 0,043 |
| Root morphology | **0,659** | **0,245** | 0,324 |
| Maximum canopy height | **-0,335** | **0,263** | -0,026 |
| Woodiness | **-0,248** | **0,365** | 0,022 |
| Vegetative reproduction | **-0,248** | **0,365** | 0,022 |
| Growth rate | **-0,248** | **0,365** | 0,022 |
| Nitrogen content | **-0,248** | **0,365** | 0,022 |
| Wood density | **-0,232** | 0,091 | -0,040 |
| Bark thickness | -0,111 | -0,118 | -0,058 |
| Leaf water content | -0,111 | -0,118 | -0,058 |
| Diameter at breast high | **-0,248** | **0,365** | 0,022 |
| Storage organs | **0,659** | **0,245** | 0,324 |
| Eigenvalue | 0,107 | 0,066 | 0,033 |
| Percentage variance explained | 46,661 | 29,035 | 14,543 |
| Cumulative % variance explained | 46,661 | 75,697 | 90,239 |
| Total inertia | 13,977 | 8,698 | 4,356 |

- **Invertebrates** group

|  | Axis1 | Axis2 | Axis3 |
| --- | --- | --- | --- |
| *Dependent variables (ecosystem services)* |  |  |  |
| Habitat for species | -0,155 | **-0,425** | -0,194 |
| Erosion control | -0,024 | -0,109 | -0,030 |
| Water purification | -0,044 | **0,218** | -0,121 |
| Pest control | **-0,265** | -0,069 | **0,334** |
| Pollination | -0,163 | 0,142 | **0,255** |
| Nutrient cycling | **0,755** | 0,089 | 0,027 |
| Seed dispersion | -0,062 | **0,245** | **-0,206** |
| Soil fertility | **0,516** | **-0,207** | 0,117 |
| *Explanatory variables (effect traits)* |  |  |  |
| Size | 0,176 | **0,234** | 0,124 |
| Feeding habit | -0,138 | 0,086 | **-0,373** |
| Diel activity | -0,188 | **-0,293** | 0,032 |
| Dispersal activity | -0,134 | 0,078 | **-0,392** |
| Mobility | -0,105 | **-0,371** | **-0,236** |
| Foraging | **0,786** | -0,115 | 0,137 |
| Diet | 0,178 | **0,274** | -0,194 |
| Habitat dependency | **0,786** | -0,115 | 0,137 |
| Microclimate moisture preference | **0,786** | -0,115 | 0,137 |
| Pollinating | -0,109 | 0,166 | **0,202** |
| Pronotum width | **0,309** | 0,116 | -0,020 |
| Eigenvalue | 0,153 | 0,132 | 0,095 |
| Percentage variance explained | 30,455 | 26,237 | 18,965 |
| Cumulative % variance explained | 30,455 | 56,692 | 75,657 |
| Total inertia | 16,664 | 14,356 | 10,377 |

- **Vertebrates group**

|  | Axis1 | Axis2 | Axis3 |
| --- | --- | --- | --- |
| *Dependent variables (ecosystem services)* |  |  |  |
| Pest control | **-0,330** | **-0,533** | **0,406** |
| Pollination | -0,169 | -0,161 | **-0,483** |
| Nutrient cycling | **0,735** | -0,084 | 0,055 |
| Seed dispersion | -0,123 | **0,645** | **0,230** |
| Soil fertility | **0,735** | -0,084 | 0,055 |
| *Explanatory variables (effect traits)* |  |  |  |
| Size | **-0,273** | -0,140 | **-0,245** |
| Feeding habit | -0,051 | 0,059 | -0,074 |
| Foraging | -0,097 | 0,112 | -0,140 |
| Diet | **0,302** | **0,317** | **0,504** |
| Trophic level | **-0,312** | **-0,538** | **0,508** |
| Habitat dependency | **0,769** | **0,267** | 0,152 |
| Torpor | **0,979** | -0,110 | 0,057 |
| Eigenvalue | 0,309 | 0,186 | 0,115 |
| Percentage variance explained | 45,982 | 27,626 | 17,041 |
| Cumulative % variance explained | 45,982 | 73,607 | 90,648 |
| Total inertia | 40,916 | 24,582 | 15,163 |
